# Evaluation of the Metrological Performance of a Handheld 3D Laser Scanner Using a Pseudo-3D Ball-Lattice Artifact

**DOI:** 10.3390/s21062137

**Published:** 2021-03-18

**Authors:** Rémi Bonin, Farbod Khameneifar, J.R.R. Mayer

**Affiliations:** Department of Mechanical Engineering, Polytechnique Montréal, Montreal, QC H3T 1J4, Canada; remi.bonin@polymtl.ca (R.B.); rene.mayer@polymtl.ca (J.R.R.M.)

**Keywords:** 3D scanning, handheld laser scanner, metrological performance, acquisition parameters, pseudo-3D artifact

## Abstract

This paper proposes the use of a pseudo-3D ball-lattice artifact to characterize a handheld laser scanner from a metrological standpoint. The artifact allows the computation of local and global errors in measurement by using the reference-frame-independent parameters of size, form, and distance within the measuring volume of the scanner, and in a single point cloud, without the need for registration. A set of tests was performed using the whole measuring volume, and three acquisition parameters, namely the orientation of the sweeps during the scans, the exposure time, and the distance to the scanner were analyzed for their effects on the accuracy of the scan data. A composite error including the errors in measuring size, form, and distance was used as a single figure of merit to characterize the performance of the scanner in relation to the data-acquisition parameters. The orientation of sweeps did not have a considerable effect on the errors. The accuracy of the scan data was strongly affected by exposure time and its interaction with the distance at which the artifact was scanned. The errors followed a quadratic trend with respect to the distance of the artifact to the scanner. The tested scanner performed best at its manufacturer’s recommended stand-off distance.

## 1. Introduction

Handheld 3D laser scanners are becoming increasingly popular for a variety of applications [1,2,3] thanks to their short acquisition time, portability, and ease of use compared to coordinate-measuring machines (CMMs) [4]. However, because of the relative newness of portable scanners, there is a lack of studies and standards that currently prevents the user to fully exploit their potential, despite their being used in multiple fields.

Handheld 3D scanners are often based on stereoscopic vision and triangulation: the device consists of at least one but often two cameras triangulating what they are seeing and processing it to create a 3D reconstitution [5]. With the laser technology, one or several laser lines are projected on the target, and their deformation is monitored by the cameras so that the scanner can establish the spatial location of the deformed lines [6]. Once the acquisition is complete, a point cloud or a mesh representing the scanned object’s surface is generated. For the common user, validating this output usually means a brief visual check to confirm if the surface was completely scanned, and whether there were defects in the scans, like missed regions. However, industrial applications often require stringent acceptance tests, and a proper understanding of the capabilities of a scanner in terms of accuracy and precision data [7,8,9], especially when features on a part have relatively tight tolerances. In other words, a quantitative metrological evaluation is needed.

**Figure 1:**
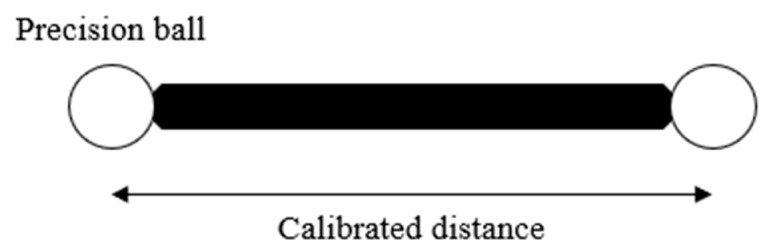
Drawing of a typical ball-bar. It allows the measurement of the two precision balls separated by a known distance.

While international standards exist to evaluate the performance of noncontact probes on a coordinate-measuring machine (CMM) [10,11], there is no international standard to evaluate the performance of handheld 3D scanners. The only existing standard is VDI/VDE 2634 [12], which is not an international standard. The standard proposes to scan a ball-bar (Figure 1) and a flat plate in several approximative orientations inside the measuring volume [12].

The two artifacts of the standard (i.e., ball-bar and flat plate) provide information on the abilities of the scanner in measuring form, size, and distance inside its measuring volume. As discussed by Ghandali et al. [13], the whole measuring volume cannot be comprehensively and systematically evaluated when using the standard’s recommendations, because both the ball-bar and the flat plate are scanned in a limited number of positions and orientations, while the user needs to systematically characterize the scanner’s performance in its entire measurement volume. Hence, there is a need for both adapted artifacts and new procedures to better characterize 3D scanners. Various attempts at creating custom artifacts or proposing new methods have been made [6,14,15] to provide information on specific aspects of the devices. The proposed artifacts usually consist of canonical shapes such as an arrangement of cylinders, planes, and spheres [16], or freeform shapes [17]. Some studies address the evaluation of CMM-mounted laser scanners [18,19,20,21], while others address laser scanners integrated with measuring arms [22]. Some artifacts are dependent on a reference frame being generated as the scans progress. The scan might also be required to be aligned with the reference data or CAD model [23], or aligned with other scans to compare them, which can mix the errors being evaluated with the alignment errors. Genta et al. [24] have used a 2D ball-plate as a reference artifact for calibrating a laser scanner. They measured the coordinates of the centers of precision balls of the artifact once with a contact probe on CMM and then with the scanner. Then, they assessed the performance of the scanner based on the difference between the coordinates of the ball centers obtained from the two measurement sets. The comparison of the coordinates of the ball centers requires registration to bring the two data sets in a common coordinate frame, which can increase the uncertainty of the assessment method.

**Figure 2:**
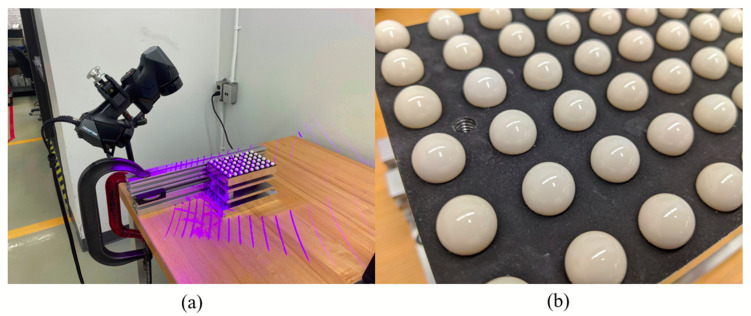
(**a**) HandySCAN BLACK scanning the pseudo-3D artifact. Note that this is only for illustration, as the scanner is not perpendicular to the table, which differs from the setup described in Section 2.4.1. (**b**) Close-up picture of the calibrated spheres of the ball-plate.

Ghandali et al. [13] extended the idea of using a CMM-calibrated ball-plate and proposed a pseudo-3D ball-lattice artifact (Figure 2) and a novel data-analysis method for evaluating the metrological performance of structured-light 3D scanners based on the calibrated reference values of the artifact. By combining calibrated spheres located in a pseudo-3D space, errors in measuring center-to-center distances, diameters, and forms of the spheres can be computed without the need for the registration of the scan-coordinate frame onto that of the CMM reference data. The artifact then provides information on several errors, locally and globally, at the same time. The original study investigated a structured-light scanner to demonstrate the use of the artifact.

This paper investigates the use of the pseudo-3D artifact for a handheld laser scanner, which is a category of laser scanners underrepresented in the literature compared to the scanners mounted on CMMs. Compared to structured-light scanners, one important difference to consider is the need to move the laser scanner relative to the artifact, which involves new parameters nonrelevant for structured-light scanners. Using the artifact’s recommended framework for a laser scanner also requires special precautions because the scanner is handheld. While the results presented in this paper are specific to handheld laser scanners, the discussed precautions and recommendations on how to use the artifact are also applicable to scanners on CMMs and robotic arms, thanks to the methods used.

## 2. Materials and Methods

The original procedure for evaluating the metrological performance of a 3D scanner using the pseudo-3D artifact [13] focused on a single-scan scenario for structured-light scanners. Here, it will be expanded to several tests for a number of acquisition parameters, in order to identify the best practices to use the artifact for characterization and validation of handheld laser scanners. The artifact allows for the study of the error in measuring the diameters and the forms of spheres, and center-to-center distances between the spheres, with no need for alignment (roto-translation) of any sort. This core property of the artifact remains true while using a handheld laser scanner. The errors computed are then used as parameters to assess the accuracy of the scanner.

### 2.1. Scanner

The laser scanner being evaluated is the HandySCAN BLACK|Elite by Creaform. It is a handheld scanner projecting two sets of 11 blue laser lines (Figure 2), creating lozenges by intersection, in which deformation is recorded and triangulated by two cameras. It uses reference targets to position itself relative to the scanned object. The targets are stickers that the users can stick on the object’s surface or its near surroundings inside the scanner’s field of view. These markers should not be removed or displaced during the acquisition, because the scanner’s data acquisition software uses the measured location of the markers to estimate the scanner’s position relative to the object at all times during a scan, and to align each scan with the following one. More details of the scanner specifications are presented in Table 1.

### 2.2. Artifact and CMM Calibration

**Figure 3:**
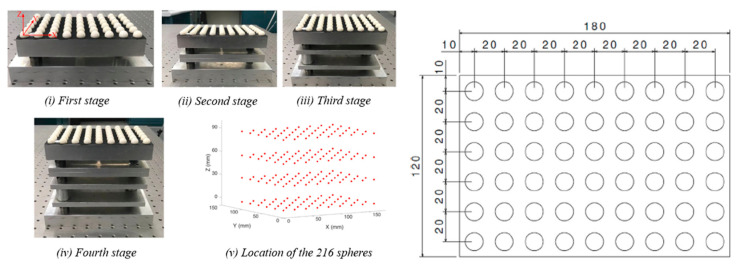
Pseudo-3D ball-lattice artifact being assembled to create a scannable 3D grid, and drawing of the ball-plate, sitting on top of the baseplate and the spacers [13]. The dimensions are in mm.

The pseudo-3D ball-lattice artifact provides repeatable positioning in 3D of 54 precision balls of the ball-plate (Figure 3). Data that can be acquired from the artifact consists in the location, form and size of these calibrated spheres. The ball-plate can be measured at four stages of different heights, resulting in the pseudo-3D grid of 216 spheres.

As shown in Figure 3, at the first stage, the artifact sits directly on the baseplate via kinematic couplings without the use of any spacer. The other three stages from the second to the fourth stage are obtained by mounting the same ball-plate on top of spacers. A spacer of 30 mm nominal height and a spacer of 60 mm nominal height create the second and the third stage, respectively, and stacked together they generate the fourth stage. The use of kinematic couplings ensures the repeatability of the assembly. As an improvement over the original design, magnets were added inside the kinematic couplings linking the baseplate, the ball-plate, and the different spacers in order to avoid any loss of the kinematic contacts during the CMM calibration, as the touch-trigger probe applies a significant force along Z-axis of the probe. Other details of the artifact design can be found in [13].

The calibration of the artifact consists of recording the position of the centers of the spheres, which will be used to obtain the reference data for center-to-center distances between the spheres. The uncertainty associated with the CMM measurements was investigated for the original CMM calibration in [13]. The uncertainty, a few micrometers at most, was at least one order of magnitude inferior to the accuracy of scanner data, which allowed the assumption that the CMM calibration data can be used as a reference for the measured distances. A new, more recent calibration was performed and used for the computations presented here. The same artifact, with the added magnets, was calibrated. Details about the equipment used are presented in Table 2. The CMM used was a Zeiss Prismo, with a Zeiss Ruby stylus. A 33 mm stylus using a 60 mm extension was mounted on the Zeiss Vast Gold probe. The ball tip had an effective radius of 1.5014 mm. Each sphere was measured twice at 5 points on its surface: the first measurement served to correct the second one, which was used for the computations. The five resulting points were used in a least-square fitting performed by the CMM software, which minimized the squared sum of the fitted residuals.

The spheres were precision partial balls manufactured by Bal-Tec. They had a nominal diameter, *d_ref_*, of 12.7 mm, and the supplier reported a sphericity of 0.64 μm and a diameter variation of 0.64 μm.

### 2.3. Methodology for Metrological Evaluation

**Figure 4:**
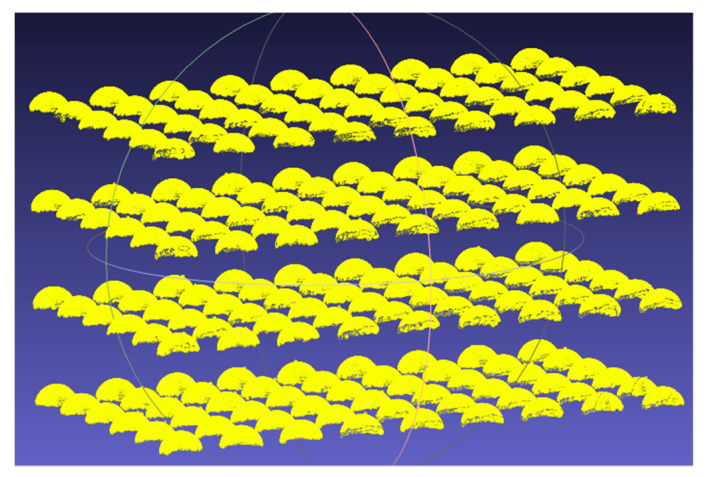
Point cloud obtained with the HandySCAN BLACK after removing undesired points from the table and parts of the artifact that are not the spheres.

Once the artifact was scanned by the scanner, the initial point cloud was trimmed to remove the undesired areas of the artifact and only retain data from the spheres’ surfaces, as shown in Figure 4. A sphere was then fitted individually to each sphere’s data points: the 3 coordinates of the center and the radius were approximated by a linear least-square fit, and these estimates were used as an initial guess for the nonlinear solver of SciPy in Python, which computed, at each iteration, the squared sum of residuals, and tried to find the global minimum until the objective function varied less than $1\times 10^{-12}$ between two consecutive iterations.

**Figure 5:**
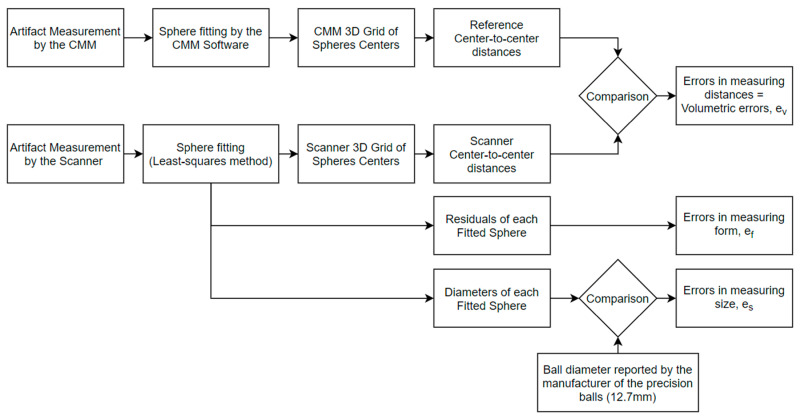
Overview of the measurement process and data analysis for the error parameters.

Figure 5 presents the step-by-step measurement process and details on how the different datasets were compared.

#### 2.3.1. Volumetric Errors

Error in measuring distances was used as a global error to characterize the volumetric accuracy of the scanner inside its measuring volume. The distances used were the center-to-center distance between the spheres of the artifact. The coordinates of the ball centers were obtained by CMM measurement. The disposition of the 216 spheres means that 216 center points were available to compute 23,220 unique center-to-center distances (143 different nominal distances) inside the field of view of the scanner. The same center-to-center distances were also measured based on the scan data and compared with the corresponding reference values of CMM measurement to estimate the errors in measuring distance. When scanning the artifact, the scanner remained at the same distance and orientation relative to the first stage of the artifact as the stages were stacked and scanned. This was done to easily repeat the measurements under the same conditions.

The center-to-center errors in measuring distance, $e_{distance,\;i}$, were then plotted against the nominal distances. The largest absolute error was used as a single value representing the volumetric accuracy of the scanner at its worst in order to simplify the interpretation of the test. In this case, the maximum of all absolute errors in measuring distance was chosen as the indicator of the volumetric accuracy, $e_{v}$:

(1) $$e_{v}=\max\left(\left|e_{distance,\;i}\right|\right)\;$$

Results and discussion are presented in Section 3.1.1.

#### 2.3.2. Errors in Measuring Size and Form

The error in measuring size, $e_{s}$, was assessed as the difference between the diameter of the sphere fitted to the scanner’s point cloud for each ball,$\;d_{scan}$, and the ball’s diameter reported by the manufacturer of the precision balls,$\;d_{ref}$, as in Equation (2). Depending on the device, the scan data could be overestimating or underestimating the diameter of each ball.

(2) $$e_{s}\;=d_{scan}-d_{ref}$$

The residual of each scanned data point from the fitted sphere was used to evaluate the random component of error in measuring form. Ghandali et al. [13] observed that the distribution of the residuals, for the scanner tested in that work, followed a Gaussian law. Other researchers have used the standard deviation of residuals of scanned point clouds of planar artifacts from a fitted plane as an indicator of the noise in the scan data [18,27,28].

**Figure 6:**
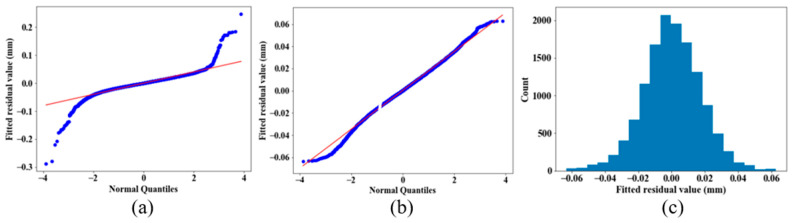
(**a**) Unfiltered-residuals Q–Q plot; (**b**) filtered-residual Q-Q plot; (**c**) histogram of filtered residuals. For the filter, the absolute fitted residuals more than 3 times the standard deviation of the whole population were considered outliers and removed.

Verification of the assumption of normal distribution was obtained in this work by plotting the residuals in a normal quantile plot (also known as a Q–Q plot) [29], which helped us judge the likeness to a Gaussian distribution, and by plotting the histogram of the fitted residuals. Figure 6 shows one typical example of the distribution of the fitted residuals.

The residuals appeared to follow a Gaussian distribution, with noticeable outliers appearing in the tails of the plot (Figure 6a). For this verification only, a simple outlier removal was made, using the same data set, based on the standard deviation of the fitted residuals: if the absolute value of the residual was more than 3 times the standard deviation, it was removed. The 3-sigma threshold means that around 99.7% of the points should theoretically be kept in a point cloud of a sphere to be used for the fitting algorithm. During the computations, after filtering the raw data with this approach, 98.9% of the points on a sphere were kept on average, with a minimum of 98.3% and a maximum of 99.9%. Figure 6b,c show the Q–Q plot and the histogram of the filtered residuals of the same case, respectively.

Except for this normality check, for all the computations in this study, the residuals were not altered in any way (save for the inevitable software processing done by VXelements, the HandySCAN BLACK software), no filter was applied, nor any outlier removed.

As proposed in [13], the error in form measurement, $e_{f}$, was computed using 3σ, meaning 3 times the standard deviation σ of the fitted residuals, to prevent outliers from interfering with the results. The error in form measurement was thus calculated as follows:

(3) $$e_{f}=3\cdot \sigma \;=\;3\cdot \sqrt{\frac{1}{\left(n-1\right)}\sum_{i=1}^{n}{\left(\delta_{i}-\bar{\delta }\right)}^{2}}$$

where $\delta_{i}$ is the fitted residual, $\bar{\delta }$ is the mean of the fitted residuals, and *n* is the number of fitted residuals. The mean of the fitted residuals should be 0 if the least-squares fitting is performed well. In practice, it was less than $1\times 10^{-10}$ mm.

Results and discussion on these local errors in measuring size and form are presented in Section 3.1.2.

#### 2.3.3. Composite Error

A composite error, based on the three errors previously described, was proposed to have a single figure of merit summarizing the results:

(4) $$e_{c}=\sqrt{\frac{1}{3}\left(e_{s}{}^{2}+e_{f}{}^{2}+e_{v}{}^{2}\right)}$$

This error should be accompanied by a mention of the radius of the spheres, as the effect of the spheres’ size on the errors has not been yet studied. For now though, it will remain $e_{c}$ and not $e_{c_{12.7}}$ for instance, the subscript indicating the diameter used.

The composite error was complementary to the three individual errors, and all those metrics were studied independently to draw conclusions. Moreover, the errors used in the computation were the absolute maximum, minimum, and mean errors for each category, to show the two extremes for the composite error, $e_{c_{max}}$ and $e_{c_{min}}$, and the mean composite error $e_{c_{mean}}$. We used the composite error to evaluate the effects of the scanning parameters on the accuracy of scan data (see Section 3.2).

To assess the variation of the composite error, a pooled standard deviation was computed using the standard deviation of the errors in measuring size, form, and distance, using the following formula:

(5) $$\sigma_{c}=\sqrt{\frac{\left(n_{s}-1\right)\cdot \sigma_{s}{}^{2}+\left(n_{f}-1\right)\cdot \sigma_{f}{}^{2}+\left(n_{v}-1\right)\cdot \sigma_{v}{}^{2}}{n_{s}+\;n_{f}+\;n_{v}-3}}$$

where $n_{s}$ is the number of errors in measuring size, $n_{f}$ is the number of errors in measuring form, $n_{v}$ is the number of errors in measuring distances, $\sigma_{s}$ is the standard deviation of the errors in measuring size, $\sigma_{f}$ is the standard deviation of the errors in measuring form, and $\sigma_{v}$ is the standard deviation of the errors in measuring distances.

### 2.4. Experimentation

This section details the different procedures and experiments to acquire the necessary data. The experiments focused on building a global understanding of the scanner’s capabilities (i.e., its errors in measuring distances, size, and form inside its measuring volume) and the impact of three scanning parameters, namely the orientation of sweeps, exposure time, and Z-distance from the scanner, on the measurement errors.

#### 2.4.1. General Setup

**Figure 7:**
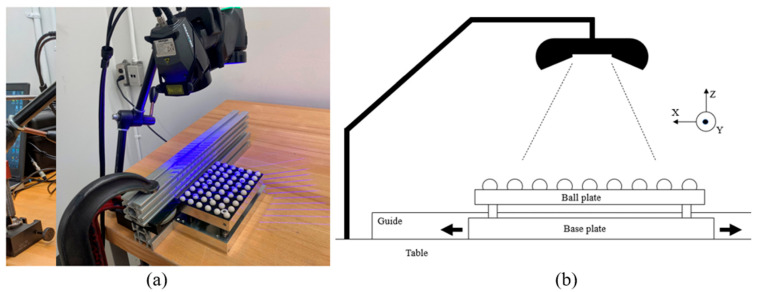
(**a**) Setup used for the scans; the guide and the scanner were fixed. The only moving part was the artifact. The scanner could be orientated differently with a ball-joint attachment (here, it is parallel to the *X* direction). (**b**) Illustration of the setup after the scanner was immobilized in its chosen orientation. The artifact was moved in and out of the field of view.

**Figure 8:**
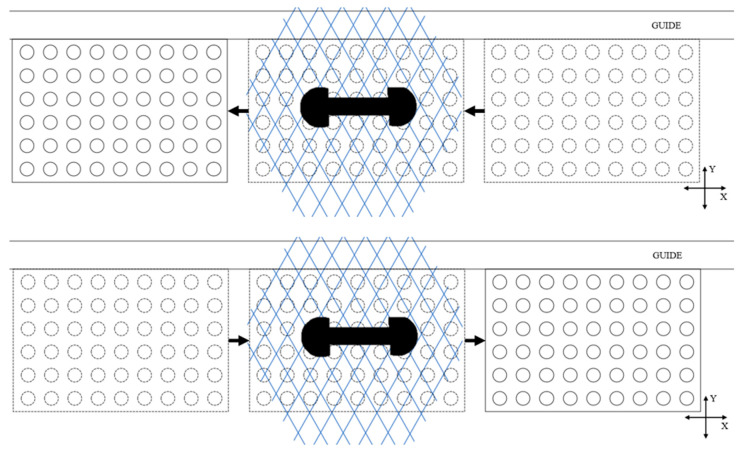
Two consecutive sweeps. The artifact moved from right to left for the first sweep. Once it left the field of view of the scanner, the acquisition was paused, and a point cloud was exported. The acquisition then resumed, a new sweep from left to right was added, and a new denser point cloud was exported. The drawing is not to scale. The scanner was actually larger than the artifact, as we can see in Figure 7a. However, to help the visualization, this drawing depicts it as smaller.

A relative movement between the laser scanner and its target is needed to acquire data. When the scanner is manipulated by a human and in a practical context, it is difficult to repeat the same scanning patterns. The user keeps an eye on the acquisition software to often arbitrarily judge if the scan looks complete and dense enough. For the tests described here, however, having a repeatable scanning pattern was sought to achieve results for comparison and validation purposes. Therefore, to be able to repeat each procedure, the proposed tests followed a prescribed scanner-motion pattern. The scanner was fixed, normal to the XY plane represented by the table on which the artifact was moved manually, guided by a straight rail, and as smoothly as possible. The scanner’s orientation relative to the artifact was parallel to either the *X* direction or *Y* direction (Figure 7). The user manually moved the artifact back and forth through the scanning area, starting outside of it, and leaving it completely for each crossing, which is called a “sweep” (Figure 8). This movement remained the same for all the scans.

#### 2.4.2. Single-Stage and Four-Stage Scans

Except for one test in this paper, all the other tests were performed using all four stages of the artifact. The test that used a single stage was the one related to the investigation of the effect of the orientation of sweeps, presented in Section 3.2.1. For the single-stage scan, the scanner was fixed in such a way that the first stage of the artifact was at the manufacturer’s recommended stand-off distance of the HandySCAN BLACK (300 mm) from the scanner. Only the first stage mounted on the baseplate was scanned. Reference targets were used on the baseplate and not on the ball-plate; the baseplate being slightly larger than the ball-plate allowed the user to stick the targets facing up near the edges of the baseplate, while still being in the field of view of the scanner when the stages were stacked.

**Figure 9:**
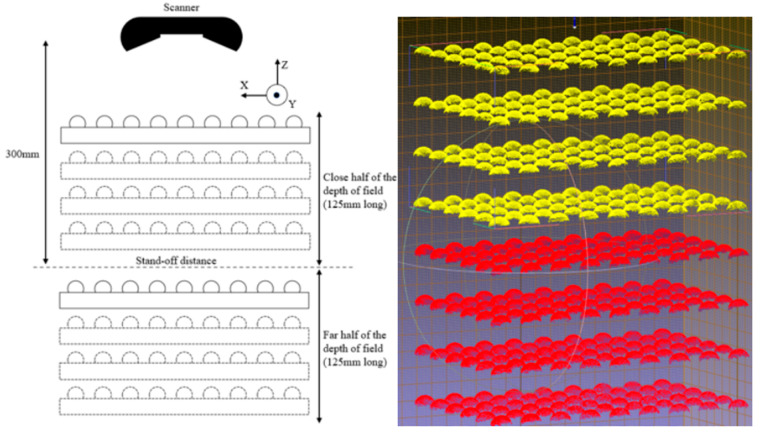
Setup for the measurements involving distance, and the resulting point clouds. The four stages of the artifact were fully scanned in two different ranges of the measuring range of the scanner. The yellow and red spheres correspond to the first and second setups, respectively.

For a global performance or validation test, as well as for the impact of distance and exposure time, the complete pseudo-3D artifact, i.e., all four stages, was used. The depth of field of the HandySCAN BLACK is 250 mm. Therefore, to cover almost the entire depth of field of the scanner, we scanned the complete pseudo-3D artifact at two locations. First, the artifact was placed in a way that the first stage was 285 mm from the scanner, meaning that the fourth stage was 195 mm from the scanner. Next, the artifact was placed in a way that the first stage was 405 mm and the fourth stage was 315 mm from the scanner. Altogether, the two scans resulted in eight stages (Figure 9) that covered almost the entire depth of field of the HandySCAN BLACK.

### 2.5. Software and Algorithms

The acquired point clouds were trimmed and merged with VXelements, the scanner’s data-acquisition software. The rest of the computations and every algorithm or fitting performed were done in Python 3 in a Spyder environment for the scanner data, and the CMM software for the CMM data. The error computations were implemented in Python. The plots were drawn using Matplotlib and Plotly.

## 3. Results and Discussion

The overall performance of the scanner inside its measuring volume and the impact of the acquisition parameters are presented and discussed in this section.

### 3.1. Accuracy of the Scanner in Its Measuring Volume

Following the procedure described in Section 2.4.2, the four stages of the artifact were scanned at the aforementioned distances from the scanner to map its full depth of field and present a clear view of the performance of the device.

#### 3.1.1. Global Errors in the Measuring Volume

**Figure 10:**
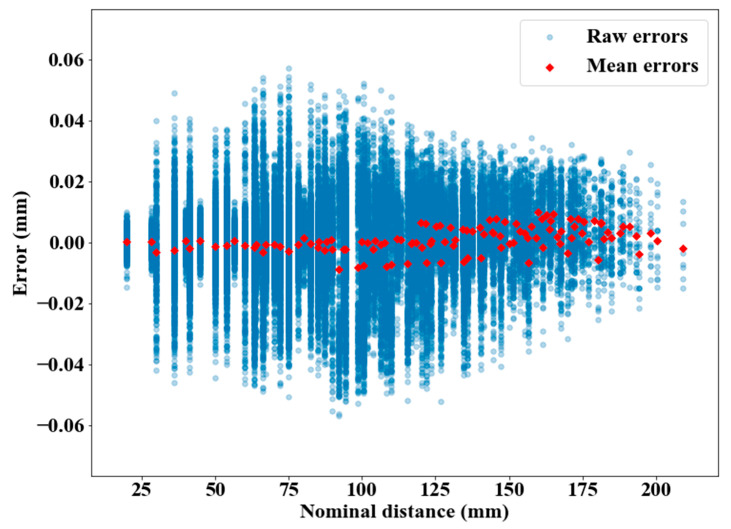
Volumetric errors of the HandySCAN BLACK based on the entire depth of field. The distances were computed inside both halves of the recommended depth of field.

Concerning the volumetric accuracy, a scatter plot can be drawn by using the two 3D grids and plotting their combined volumetric errors, which are the errors in measuring distances (Figure 10). There were 143 nominal center-to-center distance values created by the spheres of the pseudo-3D artifact, and each nominal distance had many occurrences within the artifact with various orientations and positions. Overall, errors in measuring 46,440 (i.e., 2 × 23,220) distances were plotted as the four stages were scanned at two positions. The distances did not involve spheres belonging to different setups, simply because those distances were not calibrated.

**Figure 11:**
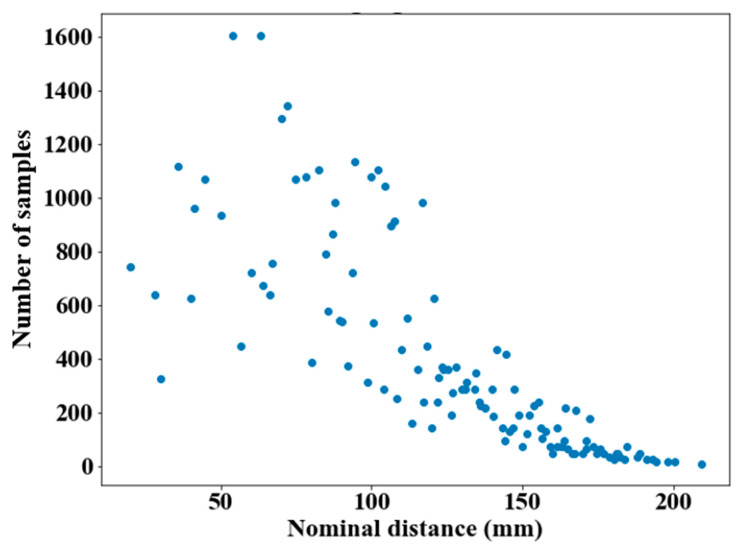
Number of samples for each nominal distance.

No trend seemed visible at first, and the errors fluctuated around 0, which means that the scanner almost equally overestimated and underestimated the measured distances in its measuring volume. The distances in the 50–125 mm range were subject to the largest variations for an identical nominal distance, which seemed consistent with the fact that they included a large portion of the total number of samples of distances (Figure 11), thus variations were more likely to occur.

Overall, this simple computation can provide an exploitable dataset for validation, testing, and possibly scanner-modeling purposes, by providing much more data than what is currently recommended in guidelines such as the VDI/VDE 2634 [12], which was originally used for the metrological evaluation of the HandySCAN BLACK by its manufacturer.

The mean of absolute errors and the standard deviation of errors of distance measurement for all 46,440 cases were calculated for the HandySCAN BLACK based on the corresponding data set in Figure 10, and the values were 0.009 mm and 0.013 mm, respectively.

#### 3.1.2. Local Errors

The errors in measuring size and form estimated using the artifact were the local errors in the measuring volume of the scanner. Both can be plotted for the whole measuring volume by scanning the complete artifact twice, using the two setups at different ranges, as explained in Section 2.4.2.

**Figure 12:**
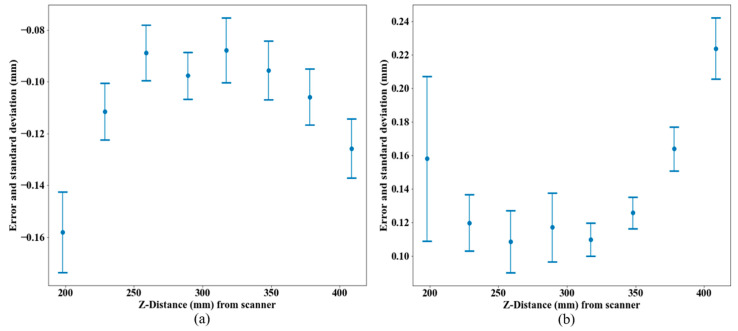
(**a**) Mean and standard deviation of errors in measuring size, and (**b**) mean and standard deviation of errors in measuring form for each stage. Error bars correspond to one standard deviation for each stage.

Figure 12 presents the mean and standard-deviation values at each stage for errors in measuring size and form.

The error in size measurement was more homogeneous across the grid than the error in measuring form, which was attributed to a higher sensitivity of the latter to outliers. An important point was to confirm that the values for each error were quite similar across the same stage so that the assumption of treating the errors at one stage as a single value in the composite error later in Section 3.2.3 is justified.

The negative error values in Figure 12a revealed that the scanner underestimated the diameter of the spheres. Figure 12 shows that both errors had smaller absolute values at the stages located near the manufacturer’s recommended stand-off distance (300 mm). The next section will present more results concerning different scanning parameters, including the Z-distance range.

### 3.2. Effects of Scanning Parameters

In order to identify good practices while using both the artifact and the reviewed laser scanner, the effects of the orientation of sweeps, exposure time, and Z-distance were investigated.

#### 3.2.1. Orientation of Sweeps

The orientation of sweeps must be considered as an acquisition parameter for the laser scanner, as opposed to other technologies (e.g., structured-light scanners) that might not need a movement for scanning an object. Only the first stage of the artifact was used for examining the effects of the orientation of sweeps on the acquired data. This test helps choose the optimal orientation to scan objects and must be one of the first tests done, as it can condition the other tests.

**Figure 13:**
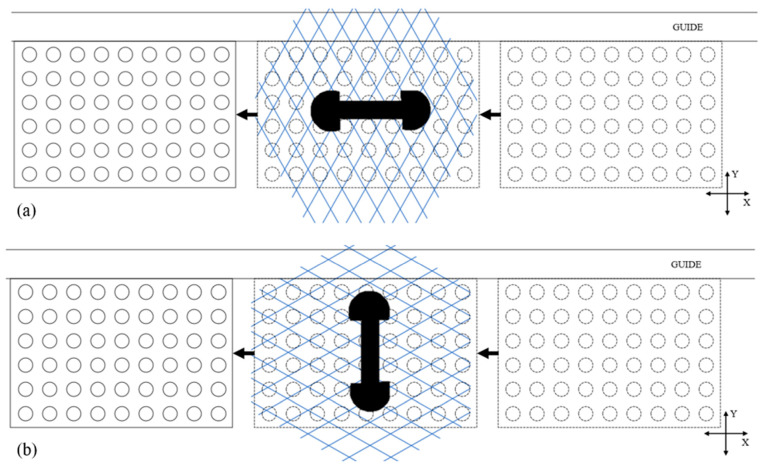
(**a**) Scan with laser scanner parallel to the *X*-direction. (**b**) Scan with laser scanner parallel to the *Y*-direction. The drawing is not to scale. The scanner was actually larger than the artifact, as we can see in Figure 7a. However, to help the visualization, this drawing depicts it as smaller.

The orientation of the scanner was defined by the line joining its two cameras. To simplify the process for the movement of the scanner, two translations, corresponding to the two orientations of Figure 13, were considered as scanning patterns: the artifact will move in a straight motion relative to the scanner, and no rotation will be involved. The X-orientation was defined by the line joining the two cameras, parallel to the longest edge of the artifact, and the *Y*-direction was orthogonal to the *X*-direction.

**Figure 14:**
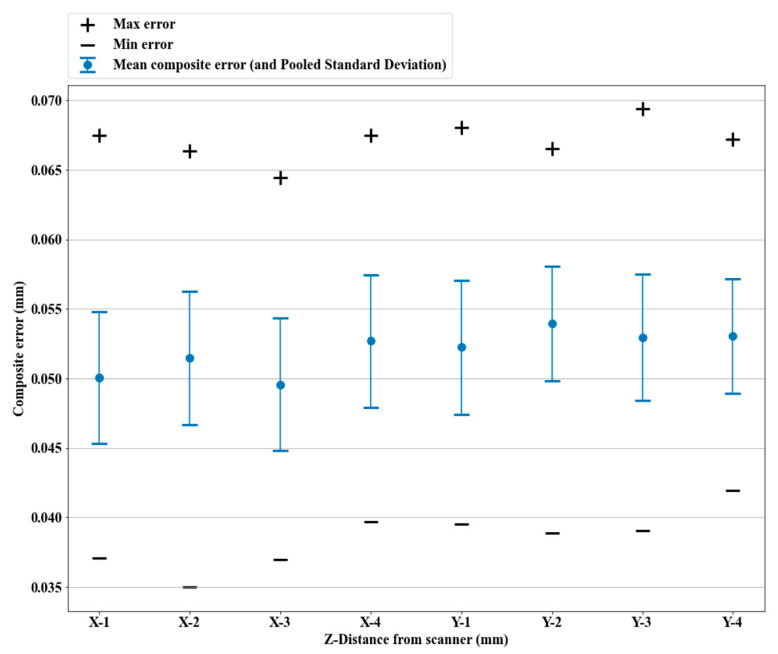
Composite error ($e_{c_{max}}$, $e_{c_{min}}$, $e_{c_{mean}}$, and $\sigma_{c}$) for the two orientations of sweeps. Repeated scans (4 sweeps) for both orientations were used to assess the spread of the results.

We scanned the artifact with four scans (each scan being one sweep) in each direction X and Y. The composite errors $e_{c_{max}}$, $e_{c_{min}}$, and $e_{c_{mean}}$ were calculated for each scan, as well as the pooled standard deviation $\sigma_{c}$, as explained in Section 2.3.3. Figure 14 shows the results for each of the four scans for the two tested orientations.

The composite error suggested a slight advantage for the X-orientation, but not significant considering the spread of results, though it did match the manufacturer’s recommendations. The laser lines were not perfectly orthogonal to each other; instead, they formed diamond shapes with acute and obtuse angles. The manufacturer recommends moving the scanner along the bisector of obtuse angles of the laser grid, which matches the results here.

#### 3.2.2. Exposure Time

A recurring acquisition parameter for laser scanners is the exposure time, which is the time interval for which the cameras allow light to hit their sensors to record an image. It effectively controls how much light energy is received.

The HandySCAN BLACK software enables the user to control the exposure time manually as well as automatically. This setting, when properly handled, might help in acquiring objects depending on their surface finish. Cuesta et al. [30] studied the impact of illumination and laser intensity of a laser-stripe system on the scanning of parts with different surface roughness, and concluded that their results on the laser-light intensity are also valid for other systems based on triangulation that replaces the intensity according to the exposure time.

The calibrated spheres of our artifact had a reflective shiny surface, which was convenient to test the impact of the exposure time. Ideally, a full study of the consequences of different surface finishes should be conducted in future work, as the results of this test may not apply to other surface finishes.

Two different exposure times were tested on the first stage of the artifact at the stand-off distance. The levels were chosen based on preliminary scans to see what the usable exposure time range for the artifact was. Exposing for more than 0.5 ms or less than 0.15 ms resulted in an unexploitable point cloud for validation purposes, especially on the top of the spheres. VXelements, the acquisition software, automatically ignored the underexposed or overexposed points.

**Figure 15:**
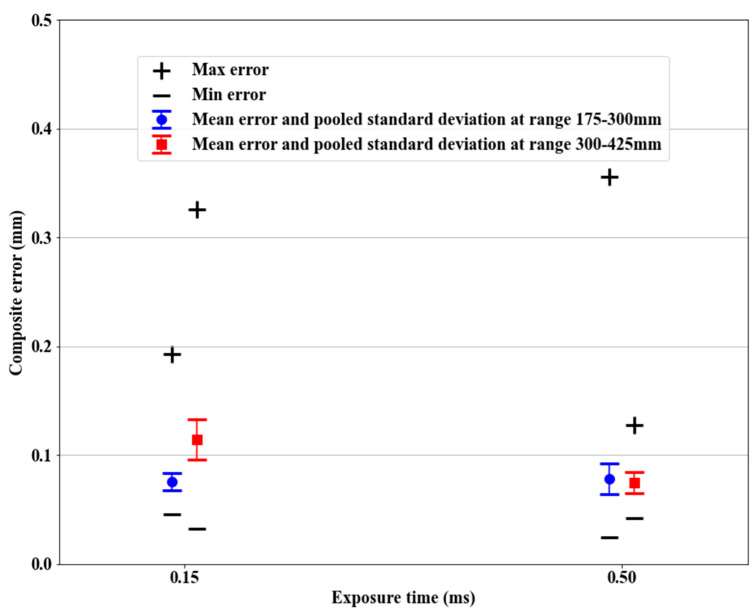
Composite error ($e_{c_{max}}$, $e_{c_{min}}$, $e_{c_{mean}}$, and $\sigma_{c}$) vs. exposure time. The four stages of the artifact were scanned. An adequate exposure time for different ranges helped to reduce the errors. For visibility purposes, the points were slightly offset from their 0.15 and 0.5 values.

The full artifact was scanned in the two different halves of the measuring volume: the close half, ranging from 175 mm to the stand-off distance at 300 mm, and the far half, ranging from 300 mm to 425 mm. The two exposure times (0.15 ms and 0.5 ms) were tested for each range, and the mean and maximum composite errors were computed and compared. The composite errors (as shown in Figure 15) highlighted an interaction between the distance to the scanner and the exposure time.

The composite errors overlapped for the two halves, but a longer exposure time helped to reduce the errors for the far half of the measuring volume, while it increased the range of errors in the close half. Scanning at different heights required a specific exposure time to achieve less variability across the measuring volume, and to reduce the expected maximum errors. This interaction might be increased by the shiny and reflective surface of the spheres, but should be considered for all kinds of surfaces. More levels of exposure time should be tested, should the application need it, while considering the surface finish.

#### 3.2.3. Z-Distance

During the tests, the assumption was that the error at each stage could be treated as a single value affected mainly by the distance from the scanner; we saw in Figure 12 that in terms of the error in measuring size and form, no large discrepancy between the spheres of one stage could be observed. This point was detailed in Section 3.1.2.

For this experiment, the two scans of the artifact covering the depth of field of the scanner were acquired with different, appropriate exposure times to achieve sound results, as discussed in the previous section. The HandySCAN BLACK has a high dynamic range (HDR) mode that helps adequately illuminate the overexposed or underexposed areas in the same scan. It was not used for this study, as it would have involved a new unknown and uncontrolled parameter.

**Figure 16:**
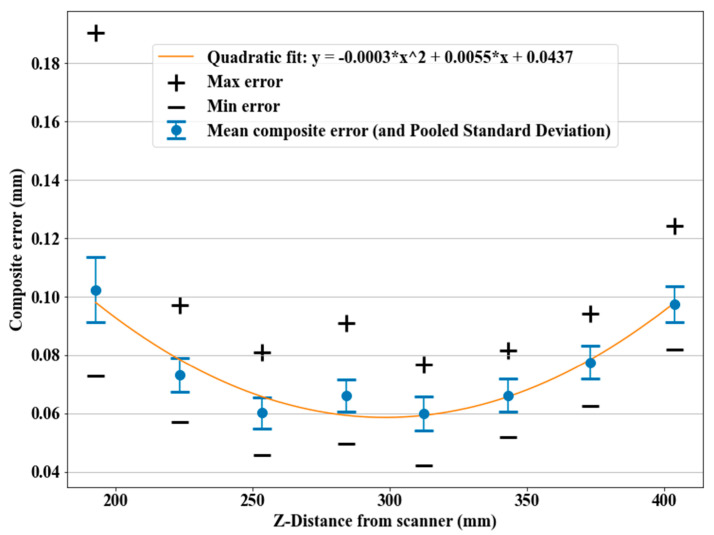
Composite error ($e_{c_{max}}$, $e_{c_{min}}$, $e_{c_{mean}}$, and $\sigma_{c}$) for each stage inside the Z-range covered by the HandySCAN BLACK, and the quadratic fit to the mean composite errors.

Figure 16 shows the mean, maximum, and minimum composite errors for each stage inside the measuring volume of the scanner. After a quadratic regression on the mean composite errors, the bottom of the parabola matched the stand-off distance specified by the manufacturer. This procedure, in combination with the artifact, seemed able to validate the performance of the scanner and pinpoint its limits.

**Figure 17:**
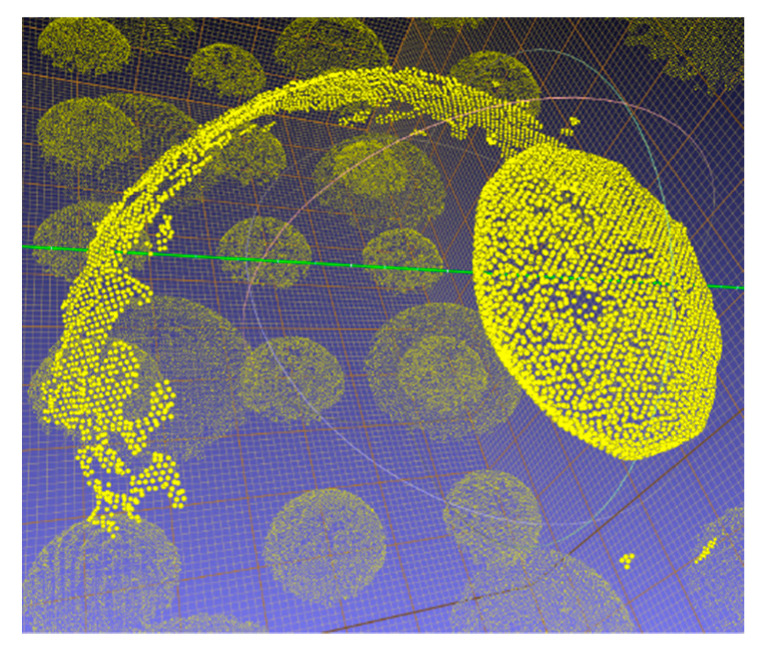
Partial scan responsible for the outlier in the composite error.

A large maximum composite error was observable on the closest stage to the scanner (195 mm Z-distance). After examining the point clouds, a poor point cloud with partial coverage of a sphere was found at that stage (Figure 17). The sphere was overexposed to the laser lines that were reflected straight toward the sensors. This resulted in the worst acquisition across the grid, which caused a poor fit and the worst result across the 3D grid.

Beyond the evaluation and the validation of the scanner, the results suggest using distances close to the stand-off distance, as being closer or farther might result in a huge increase in the errors, especially at the closest distance.

## 4. Conclusions

A new set of tests and metrics has been proposed in this paper for evaluating the metrological performance of handheld 3D laser scanners based on a pseudo-3D ball-lattice reference artifact. The artifact allowed quantifying the errors of the scanner in measuring distances, as well as size and form at different positions in its measuring volume. The benefit of using distance, size, and form is their independence from the coordinate frame of the artifact, which makes the data analysis free from the need for registration of the scan data to the reference data. The presented data analysis procedure and the proposed composite error should come in handy as a tool to assess the performance of a scanner and guide the user in obtaining the best possible results by fine-tuning the scanning parameters.

The experiments demonstrated that, for the tested scanner, the measuring distance from the scanner to the part had a significant effect on the accuracy of the scan data. Close to the stand-off distance (Z = 300 mm for the HandySCAN BLACK) was concluded to be the optimal measuring distance of the object from the scanner. The exposure time of the scans is also important, and should be appropriately chosen according to the specific distance of the scanner from the part. In general, more accurate scans can be achieved by lower exposure time when the object is closer to the scanner, and higher exposure time when it is farther from the scanner. It should be kept in mind that the results of one test might drive the user to redo another test based on the new results. In particular, testing the impact of distance will be affected by the exposure time. A proper response surface could be an answer to determine which is the best combination.

## Figures and Tables

**Table 1:** Scanner specifications given by the manufacturer [25] (picture from [26]).

Specification	Value	Picture
Measurement rate	1,300,000 pts/second	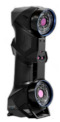
Measurement resolution	0.025 mm	
Mesh resolution	0.100 mm	
Scanning area	310 × 350 mm	
Stand-off distance	300 mm	
Depth of field	250 mm	
Recommended part size range	0.05–4 m	

**Table 2:** Equipment used for the calibration to obtain the reference data for center-to-center distances between spheres.

Specification	Model/Value
CMM	Zeiss Prismo
Probe	Zeiss Vast Gold
Stylus	33 mm + 60 mm extension + adapter plate
Ball tip radius	1.5014 mm
Probing speed	15 mm/s
Probing force	200 mN
Travel speed	160 mm/s
